# Harnessing Autophagy to Overcome Antigen-Specific T-Cell Dysfunction: Implication for People Living with HIV-1

**DOI:** 10.3390/ijms241311018

**Published:** 2023-07-03

**Authors:** Nazanin Ghahari, Roman Telittchenko, Hamza Loucif, Stephane Isnard, Jean-Pierre Routy, David Olagnier, Julien van Grevenynghe

**Affiliations:** 1 Institut National de la Recherche Scientifique (INRS), Centre Armand-Frappier Santé Biotechnologie, 531 Boulevard des Prairies, Laval, QC H7V 1M7, Canada; nazanin.ghahari@inrs.ca (N.G.); roman.telittchenko@inrs.ca (R.T.); 2 EVAH Corp., 500 Boulevard Cartier Ouest, Laval, QC H7V 5B7, Canada; ha.loucif@gmail.com; 3 Chronic Viral Illness Service and Division of Hematology, McGill University Health Centre, Glen Site, Montreal, QC H4A 3J1, Canada; stephane.isnard@mail.mcgill.ca (S.I.); jean-pierre.routy@mcgill.ca (J.-P.R.); 4 Department of Biomedicine, Research Center for Innate Immunology, Aarhus University, 8000 Aarhus, Denmark; olagnier@biomed.au.dk

**Keywords:** autophagy, Ag-specific T-cells, HIV-1, PLWH, EC, metabolism, effector functions, ART, metabolic plasticity, mitochondria

## Abstract

Like other chronic viral infections, HIV-1 persistence inhibits the development of antigen-specific memory T-cells, resulting in the exhaustion of the immune response and chronic inflammation. Autophagy is a major lysosome-dependent mechanism of intracellular large-target degradation such as lipid and protein aggregates, damaged organelles, and intracellular pathogens. Although it is known that autophagy may target HIV-1 for elimination, knowledge of its function as a metabolic contributor in such viral infection is only in its infancy. Recent data show that elite controllers (EC), who are HIV-1-infected subjects with natural and long-term antigen (Ag)-specific T-cell protection against the virus, are characterized by distinct metabolic autophagy-dependent features in their T-cells compared to other people living with HIV-1 (PLWH). Despite durable viral control with antiretroviral therapy (ART), HIV-1-specific immune dysfunction does not normalize in non-controller PLWH. Therefore, the hypothesis of inducing autophagy to strengthen their Ag-specific T-cell immunity against HIV-1 starts to be an enticing concept. The aim of this review is to critically analyze promises and potential limitations of pharmacological and dietary interventions to activate autophagy in an attempt to rescue Ag-specific T-cell protection among PLWH.

## 1. Introduction: Autophagy, a Key Degradation Program for Nutrient Recycling

Autophagy is a highly conserved and essential catabolic pathway in eukaryotic cells that is specialized in delivering cytoplasmic components and organelles to the lysosomes for digestion where hydrolases, lipases, and proteases reside. In this context, autophagy is a fundamental cellular homeostasis program that engages with harmful and/or surplus contents, such as protein aggregates (proteophagy), dysfunctional organelles including long-lived mitochondria (mitophagy), intracellular pathogens (xenophagy), and stored nutrients in lipid droplets (lipophagy). Beyond its housekeeping role, autophagy can act as a fueling metabolic program to provide sources of energy or building blocks for the synthesis of macromolecules by allowing cells to reuse its digested materials [1]. In this context, the recent role of autophagy as a metabolic contributor for cellular function and survival has received special attention in the context of cancer with tumor adaptation towards stressful environments. Originally, it is known that tumor cells preferentially consume large amounts of glucose relative to most non-transformed cells through increased glycolysis, a phenomenon described as the “Warburg effect”. This provides tumor cells with a mitochondria-independent metabolic program that allows them to quickly produce energy to sustain increased rates of proliferation and malignant progression [2,3,4]. In the Warburg effect, glycolysis terminates with L-lactate production and secretion despite the presence of oxygen. However, when glucose becomes scarce, tumor cells can rewire their metabolism to promote long-term maintenance by using alternative sources of nutrition, which is a mechanism known as “metabolic plasticity” [5,6,7]. This epigenetic process involves autophagy in providing tumor cells with recycled nutrients, that include amino acids, such as glutamine and lipid subunits, to provide additional metabolic resources rather than exclusively relying on glucose [8,9,10,11,12,13].

## 2. Thanks to Nutrient Diversification, Autophagy Becomes a New Facet of Ag-Specific T-Cell Metabolism

**Figure 1:**
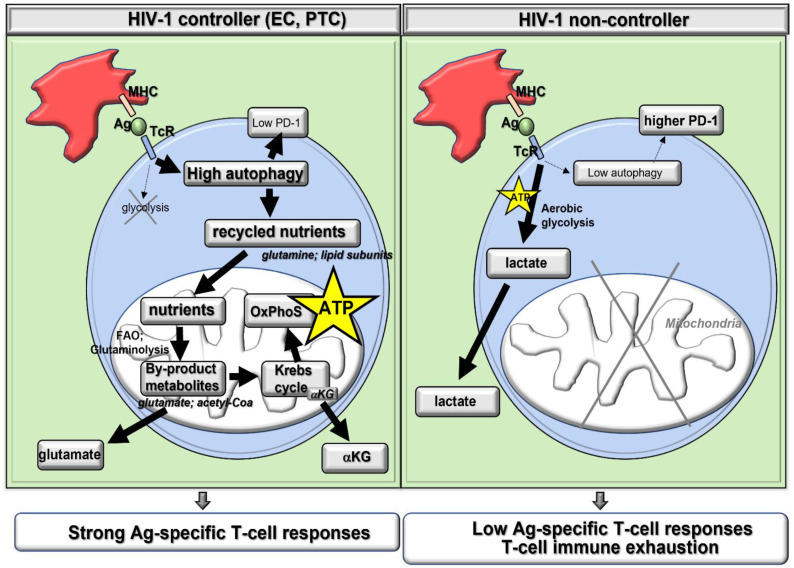
Autophagy, the driving force behind the Ag-specific T-cell immunity. Schematic summarizing how Ag-specific T-cells are using the autophagy system to diversify their nutrient sources (amino acids and lipid subunits) to ensure high energy production and robust vaccinal/antiviral-specific responses in opposition to strict glucose dependency. Of note, the figure includes the impact of autophagy on the regulation of the immune negative checkpoint PD-1 and the release of metabolite products in the extracellular plasma fluid. In red, Ag-presenting or infected cells; in blue, Ag-specific T-cells; in green; extracellular environment (plasma, ECM). ATP, adenosine triphosphate; ECM, extracellular matrix; FAO, fatty acid oxidation; OXPHOS, oxidative phosphorylation.

It is now acknowledged that autophagy-dependent metabolic plasticity in the form of recycled intracellular nutrients is not only beneficial for tumor cells, but is also used by our immune system to ensure the fast generation of effector T-cells, especially protective Ag-specific cells. First, lysosome-dependent autophagy machinery is known to be rapidly induced after T-cell receptor (TcR) engagement in the first hours of T-cell activation [14,15,16,17]. Autophagy can also be induced in vaccine-specific CD8 T-cells in healthy human volunteers, thus allowing weaker responders, such as aged individuals, to improve their immune protection [18]. Activated Ag-specific CD4 and CD8 T-cells have been confirmed to use autophagy to recycle nutrients, such as the glutamine amino acid and lipid subunits, to ensure optimal energy-dependent effector processes (Figure 1). These rely on the implementation of multiple protective programs, which include the expression of the antiviral/antitumoral cytokines (interleukin 2 (IL-2), IL-21, tumor necrosis factor alpha (TNF-α), and interferon gamma (IFN-γ)), the production of cytotoxic molecules such as granzymes and perforin in CD8 T-cell (CTL) activity, as well as T-cell polyfunctionality [15,16,19,20,21,22,23]. The latter refers to a T-cell’s ability to produce multiple molecules at the same time. Autophagy does not solely act as a metabolic contributor for effector functions in Ag-specific T-cells. In fact, it can also act as an immune check point to control (i) their proper TcR engagement by targeting several negative downstream regulators of cell activation [24], (ii) their levels of cell proliferation along with cell cycle progression [25], and (iii) their long-term maintenance [26,27,28]. Overall, autophagy is considered as a new and promising metabolic tool in the context of chronic HIV-1 infection treatment, where Ag-specific T-cell functions could be reinforced in PLWH despite viral suppression with antiretroviral therapy (ART) [29].

## 3. High Autophagy Is Key for Optimal Ag-Specific T-Cell Immunity in PLWH

### 3.1. Autophagy Grants the Metabolic Plasticity-Dependent T-Cell Protection Found in Elite Controllers (EC)

Despite the success of ART in fully suppressing viral replication in plasma, HIV-1 remains an incurable infection. A cure for HIV-1, which represents the next objective in therapeutic research, is defined as a durable control of viral replication in the absence of ART [30,31]. Therefore, natural control of HIV-1 remains one of the most promising models for a cure, since elite controllers (EC) represent a unique HIV-1-infected and ART-naïve group of individuals who are defined by replication-competent virus control along with effective and persistent Ag-specific T-cell immune responses for years [32,33,34,35]. Identifying the mechanisms contributing to HIV immune control has recently been highlighted by the International AIDS Society as one of the research priorities for the development of an HIV cure [36]. This is supported by a second case of an EC, known as the “Esperanza patient”, who presents with a spontaneous eradication of HIV-1 infection [37,38]. Recent single-cell transcriptional analysis has revealed that anti-HIV-1 CD8 T-cells from EC display a distinct metabolic program associated with a lesser reliance on the glycolytic pathway when compared to the PLWH receiving ART or not [39]. Similarly, before the loss of natural immune control over HIV-1, PLWH present with a specific metabolomic program, which is characterized by increased glycolytic metabolism and deregulated mitochondrial function [40,41]. This abnormal increase in the rate of glucose uptake in PLWH, which is similar to the cancer-related Warburg effect, can be associated with exhausted T-cell immune protection against HIV-1 [39]. In opposition, we recently found that autophagic activity, which we confirmed to be enhanced in CD4 and CD8 T-cells from EC including in their anti-HIV-1 cells [42], can provide a potent metabolic plasticity pathway by recycling protein- and lipid-based nutrients rather than exclusively depending on glucose (Figure 1). This metabolic plasticity found in Ag-specific T-cells from EC fuels the oxidative mitochondrial metabolism required for the preservation of their energy-dependent effector protection against HIV-1 [15,16,43]. Data suggest the possibility to rescue HIV-1-specific immunity in non-controller PLWH by artificially inducing their autophagy with AMP-activated protein kinase (AMPK) activator 5-aminoimidazole-4-carboxamide ribonucleotide (AICAR) with or without IL-21 supplementation [15,16]. Interestingly, AMPK activation is also required for the metabolic adaptations in Ag-specific CTL that allow for secondary effector cell generation in mice that are reinfected with *Listeria monocytogenes* [44]. Aside from its contributive role in providing Ag-specific T-cells with diverse sources of metabolites, high autophagy levels in T-cells from EC are also associated with HIV-1 containment through targeted elimination of the viral machinery by a selective process called xenophagy [42,45]. Similarly to autophagy, xenophagy can restrict HIV-1 infection in productively HIV-1-infected CD4 T-cells by selectively degrading Tat, a protein that is essential for viral transcription and virion production [46]. Using human lymphoid tissue cultured ex vivo, Pedreño-López S. et al. show that the autophagy inducer rapamycin can inhibit the level of HIV-1 DNA integration and viral replication [47]. Another study, performed on a human mucosal infection model, showed that several autophagy-inducing drugs other than rapamycin, such as carbamazepine and everolimus, can also limit HIV-1 acquisition and suppress viral replication in gut CD4 T-cells [48].

### 3.2. Autophagy Improves Antiviral T-Cell Generation by Supporting Major Histocompatibility Complex (MHC) Restricted Ag Presentation

As mentioned earlier, autophagy is functionally well equipped to isolate viral pathogens in autophagosomes and clear them out by lysosome-dependent degradation (xenophagy). Autophagosomes are double membrane-bound vesicles that enclose cytosolic constituents, including pathogens, which fuse with lysosomes to digest the encapsulated cargo during active autophagy. Accordingly, autophagy in conventional Ag-presenting cells, such as myeloid dendritic cells (DC) and macrophages, participates in the processing of many viral Ag including HIV-1-derived peptides, especially in the context of MHC Class II-restricted Ag presentation [49,50,51,52]. In this context, data show that the Ag-processing pathway for MHC Class II-restricted CD4 T-cell epitopes that have been associated with spontaneous control of HIV-1 replication begins with the endocytosis of exogenous antigens or autophagy of intracellular contents [53,54]. Using human monocyte-derived DC, results confirm that inhibition of autophagy with drugs or small interfering RNA reduces the degradation of incoming HIV-1 particles and subsequent activation of Ag-specific CD4 T-cells [55,56]. Recently, Sarango G. et al. have gone one step further and shown that the autophagy receptor TAX1BP1 (T6BP) can improve Ag presentation by MHC Class II molecules in the context of HIV-1-derived Gag peptides [57,58]. Another study in mice shows that it is possible for bone marrow-derived DC to improve the presentation of simian immunodeficiency virus (SIV) Gag peptides to Ag-specific CD4 T-cells by manipulating their autophagy system. Indeed, the authors show that, when fused to the autophagosome-associated LC3 cargo protein, SIV Gag proteins can be functionally targeted to autophagosomes, processed by autophagy-mediated degradation in their lysosomes, presented to MHC Class II compartments, and elicit effective potential CD4 T-cell responses [59]. Importantly, compared with the SIV Gag peptides alone, SIV gag-LC3 fusion antigen can induce stronger Ag-specific T-cell in vivo responses, which are characterized by an enhanced magnitude and cell polyfunctionality. Similarly, the immunization of mice with DNA Gag constructs reveals that, in comparison to HIV-1 Gag peptides delivered alone, the fusion of Gag Ag to the autophagy cargo p62 protein enhances the number of IFN-γ-producing Ag-specific CD4 and CD8 T-cells in animals [60]. Finally, investigators have known for a long time that CD4 T-cells in PLWH can sometimes present HIV-1 Ag, such as Gp120 peptide, to induce Ag-specific CTL T-cell responses [61]. In fact, recent data confirm that activated CD4 T-cells are indeed highly effective at MCH Class II-restricted presentation of an immunodominant HIV-1-derived Gag peptides along with its subsequent processing and presentation of endogenously produced Ag [62]. The authors show that activated CD4 T-cells can present HIV-1-derived Ag to Gag-specific CD4 T-cells to elicit their IL-2 and TNF-α production. There is also a good chance that, similarly to DC, the CD4 T-cell-mediated HIV-1 peptide presentation to Ag-specific T-cells might involve endogenous processing of HIV-1 materials through autophagy. In conclusion, autophagy machinery orchestrates Ag-specific T-cell immunity in PLWH by providing their cellular energy-dependent effector functions and by supporting their immune education through MHC restricted Ag presentation.

## 4. Autophagy in Ag-Specific T-Cells from PLWH Must Be Induced Together with ART (Figure 2)

### 4.1. As HIV-1 Proteins Hijack Autophagy to Block Lysosomal Degradation

In the absence of ART-mediated viral suppression, productive HIV-1 infection represents one of the best characterized systems in which autophagy can be disarmed by the virus via the development of multiple strategies preventing the sequestration and degradation of its proteins, thus leading to the establishment of a chronic infection [63,64,65]. In fact, several HIV-1-related proteins, such as Tat, Nef, and Vif, which are produced during the late stages of HIV-1 replication, inhibit autophagy in human cells, including infected T-cells [66,67,68,69,70,71]. Therefore, it will be critical to manipulate autophagy in PLWH when HIV-1 replication is suppressed by ART if we want to achieve a beneficial effect of autophagy-mediated lysosomal degradation and potentiate Ag-specific T-cell immune responses.

### 4.2. As Autophagy-Mediated Metabolism Favors Cell Infectivity and Viral Replication

Although metabolic plasticity mediated by autophagy in the form of recycled glutamine is key to ensure optimal HIV-1-specific CD4 T-cell responses, it is also involved in polyclonal T-cell activation which generates new targets for HIV-1 infection. Indeed, in addition to autophagy allowing energy-dependent IL-21 production and cell polyfunctionality in HIV-1-specific CD4 T-cells [15,43], it is now clear that glutaminolysis-dependent energy production also increases T-cell susceptibility to de novo HIV-1 infection [72]. In fact, data show that the entry of glutamine-derived carbons into the mitochondrial tricarboxylic acid cycle (TCA) supports the early steps of HIV-1 infection in naïve and memory CD4 T-cells [73]. The authors came to this conclusion by first demonstrating that CD4 T-cell mitochondrial biomass was related to their oxygen consumption. They also showed that the CD4 T-cell with the highest mitochondrial biomass had a higher percentage of infected cells following HIV-1 virion exposure. To conclude, the authors determine that glutaminolysis, one of the major catabolic pathways that fuels mitochondrial OXPHOS, is required for optimal viral infection. Similarly, if not suppressed by ART, HIV-1 alters CD4 T-cell metabolism by driving elevated mitochondrial energy production to increase its viral replication [74]. The authors further showed that the inhibition of mitochondrial energy metabolism with the drug metformin, which targets the mitochondrial respiratory chain complex-I, suppresses HIV-1 replication in both human CD4 T-cells and in vivo humanized mice. Finally, additional studies show that the early steps of HIV-1 infection, such as virus binding to CD4 or membrane fusion, allow the virus to increase the autophagocytic pathway, hence preparing the cells to be more permissive to viral infection [75,76,77]. Mechanistically speaking, data indicate that the autophagy-related gene (ATG) 9A is required for high HIV-1 infectivity in Jurkat CD4 T-cells in an Env-dependent manner [78]. Altogether, the data indicate that inducing autophagy in PLWH without ART-driven viral suppression may favor viral dissemination and higher Ag-specific T-cell infectivity.

**Figure 2:**
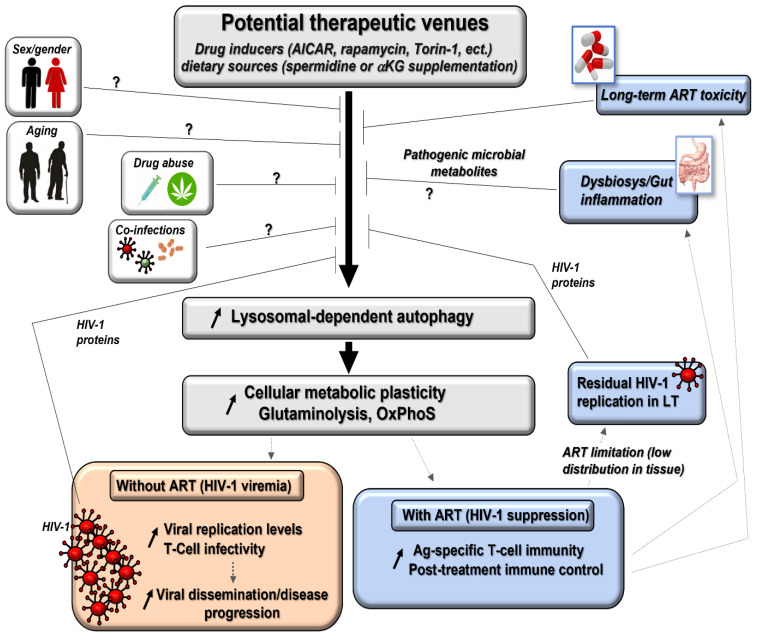
Limitations and challenges of inducing autophagy in Ag-specific T-cells in PLWH. Schematic of clinical/virological parameters that may impact our ability to efficiently harness autophagy and T-cell immune control of HIV-1 infection in the presence or absence of long-term ART. LT, lymphoid tissue. In blue, observations expected in the context of ART-induced HIV-1 suppression in treated PLWH; in red, in the context of ART-naïve PLWH and HIV-1-productive infection. ?, parameters that have been shown to impact autophagy in several research models, although not yet proven with PLWH.

## 5. Clinical Parameters in PLWH May Impact the Efficacy of Autophagy Induction with ART Co-Treatment (Figure 2)

### 5.1. Biological Sex and Age of the Individuals

Although the underlying molecular mechanisms of sex differences in autophagy remain largely unexplored, recent evidence show sex differences in autophagy in the context of cancer, inflammatory diseases, and viral infections [79,80,81]. Using bioinformatics data analyses, a 2023 study points to the possibility of autophagy as a critical contributor to sex differences in rheumatoid arthritis [82]. In an autism spectrum disorder (ASD) in vivo model, data show sex-dependent variation in transcript and protein levels for several ATG and for the autophagosome cargo p62 protein [83]. Another study in mice, which were assigned to different physical exercises, show that both biological sex and age of the animals impact the autophagy–lysosome system, with young female mice displaying greater abundance of autophagy and lysosome proteins than young males [84]. In the context of viral infection, several studies have demonstrated an influence of sex and age on autophagy. In Carp and Zebrafish that are infected with herpesvirus, data show that female fish, which are more vulnerable to the infection, have lower antiviral responses when compared to the male group [85]. The authors find that these low antiviral responses in females are mediated by increased autophagic degradation for the mediator of IRF3 activator (MITA). Furthermore, in individuals who display moderate-to-severe coronavirus disease 2019 (COVID-19), abnormal high concentration of the autophagosome cargo p62 is found in the sera of the patient, particularly in aged people who are more than 50 years old [86]. Finally, human umbilical artery smooth muscle cells in female newborns respond much better to autophagy induction with rapamycin treatment than male cells [87]. Although there is no indication of sex- and age-based differences in autophagy with PLWH, there are some proving distinct biological differences regarding protective immunity against HIV-1 between women and men, as well as in middle-aged and elderly patients. For example, the levels of immune hyperactivation in T-cells and inflammation, which are hallmarks of chronic HIV-1 infection despite ART, differ between men and women and could be a central mechanism in the sex differences observed in the rate of HIV-1 disease progression [88,89,90,91,92,93,94]. Furthermore, data show that there are sex-based differences in mortality and CD4 T-cell responses among ART-treated PLWH [95]. In this study, the authors find that women show consistently better immune responses to treatment than men and older patients. Altogether, research regarding the impact of sex and age on autophagy in the context of human viral infections including HIV-1 has just started, but it is apparent that these parameters must be considered in the context of autophagy manipulation in PLWH.

### 5.2. The Duration and Nature of Antiretroviral Drug (ARV) Regimens

While current ARV regimens are generally well tolerated, risks for side effects and toxicity remain high as PLWH must take lifelong medications [96]. In this context, using different mice, rat, and human models with peripheral blood mononuclear cells (PBMC), data show that several ARV inhibit autophagy and induce lysosome dysfunction such as the reverse transcriptase inhibitors efavirenz, zidovudine, and stavudine [97,98,99,100,101], the protease inhibitors ritonavir and lopinavir [102], and 2- to 3-drug regimens [103,104,105]. Data show that mitochondria toxicity in ART-treated PLWH are usually characterized by lower adenosine triphosphate (ATP) production, inhibition of electron transport chain complexes, impairment of fatty acid oxidation (FAO), and altered membrane potential. Since the conceptual idea of boosting autophagy is to rescue energy-dependent HIV-1-specific T-cells in PLWH, this therapeutic venue requires mitochondria to be fully functional. However, recent data show that T-cell mitochondrial functions can be impaired in PLWH by the long-term exposure to several ARV [106]. For example, it has been shown that CD4 T-cells, collected from PLWH receiving the integrase inhibitors dolutegravir or elvitegravir, showed mitochondrial respiratory impairment with lesser energy production [107]. Similarly, Maagaard A. et al. have confirmed that PLWH, which are exposed to reverse transcriptase inhibitors for years, display mitochondrial DNA loss in both CD4 and CD8 T-cells [108,109]. In summary, although the lives of PLWH are dramatically prolonged due to highly effective ART, long-term administration of these drugs have shown to impact autophagy, lysosomal activity, and mitochondrial integrity. Therefore, it may be mandatory to design and select specific ARV regimens with lesser impact on these cellular mechanisms to provide effective autophagy-dependent energy production while inhibiting HIV-1 replication. Interestingly, Angin M. et al. have recently provided a table that summarizes several drug regiments and NRTI mitochondria toxicity index which may be helpful in designing safer ART administration to PLWH [39]. Additionally, this data may also provide the initial step in our efforts to reduce ART-mediated mitochondria toxicity to a minimum in HIV-1-infected patients, therefore improving the therapeutic success of metabolism-targeted strategies via autophagy induction.

### 5.3. Low Distribution of ARV into Lymphoid Tissues

As mentioned previously, current antiretroviral therapy (ART) can achieve long-term suppression of plasma viral load to levels less than 20 copies per millimeter of plasma, especially when they are initiated early in PLWH; however, optimal ARV drug penetration in lymphoid tissues, such as lymph nodes, spleen, and gut-associated lymphoid tissue (GALT), is under debate in these individuals [110,111,112]. Data show that, despite ART, these tissues can become pharmacological sanctuaries that result in incomplete suppression of viral replication. In fact, it is believed that the poor penetration of ARV to lymphoid tissues including the mesenteric lymph nodes (MLN) may limit the therapeutic efficacy to achieve a complete viral eradication in SIV-infected rhesus macaques [113,114]. Hence, the residual HIV-1 replication in lymphoid tissues including MLN, regardless of effective viral suppression in the bloodstream, may counteract the efficacy of boosting autophagy with drugs in PLWH.

### 5.4. Changes in the Gut Microbiota

The balance of microbial communities in the gut is critical for preserving effective antiviral immune defenses, including Ag-specific T-cell immunity [115]. In fact, a group of authors found that in mice treated with broad-spectrum antibiotics to deplete gut microbiota, both antiviral CD4 and CD8 T-cells that are specific for hepatitis B virus produce lesser amounts of INF-γ, TNF-α, and IL-2 cytokines. In the context of HIV-1 infection, recent studies confirm that PLWH on ART exhibit persistent microbial gut dysbiosis, referring to a change or imbalance of intestinal flora, when compared to uninfected individuals. Recent data show that gut dysbiosis in PLWH is characterized by the depletion of commensal bacteria (*Clostridia*) and an increase of pathogenic bacteria (*Negativicutes*, *Bacilli*, *Coriobacteriia*, and *Prevotella*) [116,117,118,119,120]. In opposition, the naturally HIV-1-protected EC have richer gut commensal microbiota with unique bacterial signatures which may contribute to immune control of HIV-1 [121]. Since the gut commensal microbiota can induce autophagy, whereas pathogenic bacteria mainly suppress the autophagy flux [122,123], we cannot exclude that gut dysbiosis found in PLWH may impact the pharmacological manipulation of the catabolic pathway.

## 6. Therapeutic Tools to Induce Autophagy in T-Cells of PLWH

### 6.1. Autophagy Activator Drugs

There are many drugs available to artificially induce autophagy in human cells as well as in the context of HIV-1 infection. First, there are rapamycin, Torin-1, dactolisib, everolimus, carbamazepine, and other rapalogues that are known to enhance autophagy by inhibiting its main negative regulator; the mammalian target of rapamycin (mTor). Aside from mTor inhibition, autophagy can also be activated after induction of AMP-activated protein kinase (AMPK) by using AICAR, metformin, trehalose, and resveratrol. Although data show that in vitro induction of autophagy is responsible for the effector functions in Ag-specific T-cells, including anti-HIV-1 cells, this mainly depends on AMPK activation rather than mTor inactivation [14,15,16,124]. However, several in vivo and ex vivo observations also vouch for increased autophagy in effector T-cells using mTor-inactivating drugs. In this context, HIV-1-infected and rapamycin-treated mice show increased expression for autophagy-related proteins (ATG) including ATG-5 and the autophagy cargo microtubule-associated protein 1A/1B-light chain 3 (LC3) [125]. Ex vivo cultures of human lymphoid tissue, which is a suitable model to obtain critical insight into HIV-1 and its intricate relationship with autophagy, also confirm that rapamycin is effective in inducing autophagy in CD4 T-cells [47]. Similarly, mucosal CD4 T-cells, treated with rapamycin, everolimus, or carbamazepine in the context of an ex vivo human HIV-1 infection model, showed increased autophagy activity [48].

### 6.2. Pro-Autophagy Diets

Numerous pro-autophagic dietary interventions are being investigated for their potential therapeutic effects to enhance the protective antiviral T-cell immunity, although this therapeutic approach has not yet been thoroughly investigated in PLWH. For example, calorie restriction (CR), which refers to a chronic reduction of energy intake by 15% to 40% while maintaining an adequate intake of micronutrients such as vitamins and minerals, has been found to be effective in enhancing the proliferative response and cytokine production by Ag-specific T-cells upon reinfection with influenza virus in aged mice [126]. “Fasting”, which is a diet strategy that involves a willful abstaining from consuming nutrients for a certain period of time, is known not only to induce autophagy in leukocytes [127], but also to enhance antiviral CD4 T-cell immunity in *severe acute respiratory syndrome coronavirus 2* (SARS-CoV-2)-infected individuals through the production of ketone bodies [128,129]. Data show that ketone bodies, which can also be supplemented in the context of ketogenic diets, provide an alternative carbon source in Ag-specific CD4 T-cells to fuel mitochondrial energy production [128]. The supplementation of specific micronutrients such as spermidine, ascorbic acid (vitamin C), and niacin (vitamin B3) induces autophagy [130,131]. Spermidine, which is a natural polyamine that is critically involved in the maintenance of cellular homeostasis and acts as an anti-aging vitamin in humans, is another potent autophagy inducer [132]. In fact, the potency of spermidine in inducing autophagy has been recently quantified to be equivalent to that of rapamycin [133]. Although not available in the context of HIV-1 research, spermidine supplementation has been found to be effective in improving autophagy and vaccine-induced antiviral CD8 T-cell function in older donors [18]. Spermidine supplementation in hepatitis B virus (HBV)-vaccinated mice also improves Ag-specific CD8 T-cell protection [134]. Similarly, another recent study showed that cytomegalovirus (CMV)-specific T-cell functionality can be improved by vitamin C pre-treatment for several days [135]. Altogether, data show that while many approaches to stimulate autophagy with drugs and/or dietary supplementations, such as rapamycin, spermidine, niacin, and metformin, have shown promise in preclinical studies, their translation to HIV-1-related clinical application and their overall efficacy and safety profiles in PLWH require further investigations.

## 7. Final Remarks

Considering the growing body of evidence, manipulating the catabolic process of autophagy with drug inducers and/or specific diets is becoming a promising avenue in rescuing defective HIV-1-specific T-cell responses along with a better control of HIV-1 viral replication. Not only recent in vitro studies prove that autophagy acts as a metabolic catalyzer of Ag-specific effector functions [15,16,39], but in vivo observations also confirm that drug-mediated autophagy induction improves antiviral responses during HIV-1 infection. For example, in HIV-1-infected humanized mice, results confirm that treatment with the autophagy inducer rapamycin significantly improves Ag-specific T-cell responses. The authors further show that in vivo co-treatment with rapamycin and ART leads to significantly reduced viral rebound after ART withdrawal in infected animals [125]. It is also worth noting that, whereas the majority of PLWH experience rapid viral rebound after ART interruption, a rare population of individuals, termed post-treatment controllers (PTC), demonstrate sustained viral suppression ranging from several months to a couple of years after ART cessation [136]. Similarly to EC displaying high autophagy, metabolomic analysis of PTC show several plasma metabolites that are associated with better HIV-1 control after ART cessation; these include increased levels of glutamate and alpha-ketoglutarate (αKG), and lower levels of L-lactate when compared to PLWH with lesser HIV-1 control [137]. Overall, the literature indicates that drug-induced autophagy must be considered in PLWH before ART cessation to improve their post-treatment control of HIV-1 due to a better organized metabolic plasticity and energy-dependent Ag-specific T-cell protection. Finally, autophagy-dependent metabolic reprogramming in CD4 T-cells may also be considered to purge HIV-1 reservoirs, which persist in PLWH despite ART and reignite systemic viral replication should treatment be interrupted. This HIV-1 reservoir is primarily persistent in long-lasting memory CD4 T-cells that are latently infected with the virus [138]. Recent studies indicate that the cellular metabolism and the energy-dependent processes are major determinants of HIV-1 reservoir seeding in CD4 T-cells and may be important targets for new therapeutic approaches against HIV-1 [139,140]. In conclusion, although autophagy is a new therapeutically targetable process in PLWH, it is mandatory to improve our understanding of this mechanism in humans to better harness the potential of autophagy manipulation in HIV-1 care.

## Data Availability

No applicable.
